# Efficacy and safety of intensive antihypertensive strategy in patients with low diastolic blood pressure: a secondary analysis of a cluster randomised trial

**DOI:** 10.1136/heartjnl-2025-326413

**Published:** 2025-12-09

**Authors:** Ziyi Xie, Chuan Yang, Yangzhi Yin, Chuning Shi, Danxi Geng, Chang Wang, Ning Ye, Lixia Qiao, Nanxiang Ouyang, Linlin Zhang, Pengyu Zhang, Zhaoqing Sun, Yingxian Sun, Guozhe Sun

**Affiliations:** 1 Department of Cardiology, The First Hospital of China Medical University, Shenyang, Liaoning, China; 2 Department of Cardiology, Shengjing Hospital of China Medical University, Shenyang, Liaoning, China

**Keywords:** Cardiovascular Diseases, Hypertension, Treatment Outcome

## Abstract

**Background:**

Intensive blood pressure (BP) control <130/80 mm Hg could bring additional benefits for cardiovascular disease (CVD). However, it is unclear whether intensive BP control remains effective and safe in the general hypertensive population with low diastolic BP (DBP) since a J-shaped relationship between DBP and CVD was observed.

**Methods:**

This is a post hoc analysis of the China Rural Hypertension Control Project. Mixed-effect Cox proportional regression and generalised estimating equation models were used to determine HRs of outcomes by intensive BP control, stratified by baseline DBP quartiles. The interaction between intervention and DBP levels was assessed.

**Results:**

A total of 33 288 participants were divided into four categories according to baseline DBP quartiles (Q1–Q4): DBP ≤80.7 mm Hg, 80.7–87.3 mm Hg, 87.3–94.3 mm Hg and >94.3 mm Hg, respectively. Compared with usual care, the intervention group reduced cardiovascular outcomes across DBP quartiles with adjusted HRs as follows: Q1: 0.69 (95% CI 0.58 to 0.83, p<0.001), Q2: 0.60 (95% CI 0.50 to 0.71, p*<*0.001), Q3: 0.59 (95% CI 0.49 to 0.71, p*<*0.001) and Q4: 0.67 (95% CI 0.56 to 0.78, p*<*0.001). There was no evidence of interaction between DBP quartiles and groups (p*=*0.987). The intervention group had a higher risk of hypotension, which was observed when stratifying by different baseline DBP levels. Intensive BP reduction did not increase the occurrence of injurious falls, syncope or adverse kidney outcomes.

**Conclusions:**

The intensive BP intervention led by non-physician providers is both effective and safe in reducing CVD and mortality across all baseline DBP groups.

**WHAT IS ALREADY KNOWN ON THIS TOPIC**

- Although intensive blood pressure (BP) control (<130/80 mm Hg) can reduce cardiovascular risk, there are still doubts about its safety in patients with low diastolic BP (DBP).

**WHAT THIS STUDY ADDS**

- This study confirms that intensive antihypertensive treatment can safely and effectively reduce cardiovascular risk in all types of patients with baseline DBP (including low DBP), without adverse effects such as syncope, falls or renal injury, and is also applicable in rural, non-physician-led medical settings.

**HOW THIS STUDY MIGHT AFFECT RESEARCH, PRACTICE OR POLICY**

- This study supports widespread promotion of intensive BP management, including in people with low DBP, and confirms the feasibility of non-physician implementation in resource-limited settings, providing evidence for global hypertension prevention and control strategies.

## Introduction

 Cardiovascular disease (CVD) ranks among the leading causes of morbidity and mortality worldwide, significantly contributing to the global disease burden and posing a substantial public health challenge.^1 2^ Hypertension, as a major modifiable risk factor for CVD and all-cause mortality, is particularly prevalent in low- and middle-income countries,^3^ highlighting the urgent need for targeted interventions.

High-quality randomised controlled trials have demonstrated promising outcomes from intensive blood pressure (BP) management with the target lower than 140/90 mm Hg.^4 5^ Consequently, the 2017 American College of Cardiology/American Heart Association guidelines recommended intensive BP lowering to systolic BP (SBP) level <130 mm Hg. However, the effectiveness and safety of intensive BP lowering in patients with lower diastolic BP (DBP) is controversial.^6 7^ A large clinical cohort study showed that DBP of 80–90 mm Hg is linked to the lowest CVD risk in the general population.^6^ Results from the Ongoing Telmisartan Alone and in Combination With Ramipril Global Endpoint Trial (ONTARGET) and Telmisartan Randomized AssessmeNt Study in ACE iNtolerant participants with cardiovascular Disease (TRANSCEND) trials indicated that for high-risk groups, the optimal DBP range is 70–80 mm Hg but a DBP <70 mm Hg can elevate CVD risk.^8^ Previous studies have shown that low DBP is associated with an increased risk of CVD^8–10^ and all-cause mortality rate,^8 11 12^ both of which exhibit a J-shaped curve. Thus, DBP is a key issue when conducting intensive BP control. However, it is reported that the J-shaped relationship between baseline DBP and CVD risk might be due to confounding factors and selection bias,^13 14^ indicating that low DBP may not increase the adverse effects of BP lowering.

The 2017 American College of Cardiology/American Heart Association guidelines recommended implementation studies that demonstrated the practicality of intensive BP interventions in resource-constrained settings were needed.^15^ In rural areas, the overall level of medical care remains at a relatively low level, and there is insufficient safety monitoring of intensive BP control. The China Rural Hypertension Control Project (CRHCP), in a more representative and larger-scale sample, demonstrated that intensive BP management, with a target of <130/80 mm Hg under the care of a non-physician community healthcare provider, could significantly improve CVD outcomes.^16^ But in this real-world setting, whether the observed association between low baseline DBP and an increased risk of CVD is causal or merely an apparent phenomenon owing to confounding factors such as underlying diseases or age remains uncertain. Therefore, we conducted a secondary analysis of the CRHCP data to explore the effectiveness and safety of the CRHCP’s intensive BP control strategy in low DBP hypertensive patients in the general population, and to clarify whether the effectiveness of the multifaceted BP intervention strategy differs according to baseline DBP levels.

## Methods

This was a secondary analysis of CRHCP data. The design of the CRHCP has been described in detail previously.^16 17^ The CRHCP study was an open-label, blind endpoint and cluster randomised two-stage trial in 326 villages in three rural provinces of China (Liaoning, Shanxi and Hubei). The trial progress was monitored by an independent data and safety monitoring committee, and the safety and effectiveness of the data were examined during the trial.

### Study population

All 33 995 participants (aged ≥40 years) in the CRHCP were enrolled in this study; of these, 162 participants did not participate in the outcome collection after 3 years. After excluding 114 participants with missing biochemical indicators, 384 with missing behavioural habit indicators and 47 with missing anthropometry indicators, finally, a total of 33 288 participants were analysed.

### Randomisation and masking

Randomisation occurred at the Tulane University Translational Science Institute in the USA, employing SAS software for stratification by provinces, counties and towns. A biostatistician allocated all participating villages to either intervention or usual care groups in a 1:1 ratio according to the predetermined random allocation sequence. Randomisation assignments remained concealed until the completion of recruitment and baseline data collection. Both the members of the endpoint adjudication committee and the event adjudication coordinators were blinded to the study assignments.

### Stratification and grouping

**Figure 1:**
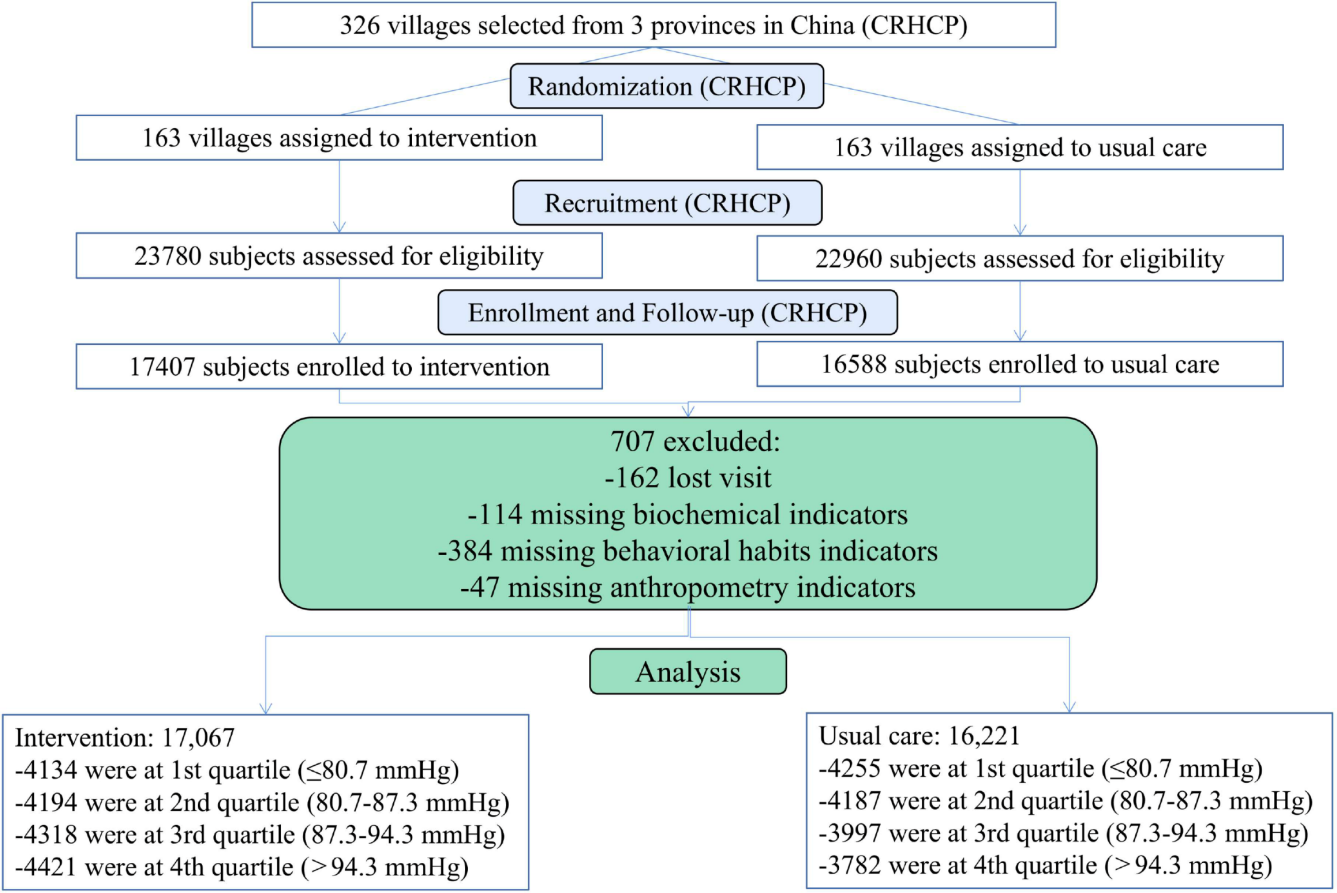
The flowchart of the study. Randomisation, recruitment and enrolment in this study is shown. CRHCP, China Rural Hypertension Control Project.

In the analysis, 33 288 participants were first stratified into quartiles (Q) according to different baseline DBP levels: the first baseline DBP quartile was ≤80.7 mm Hg, the second was 80.7–87.3 mm Hg and the third and fourth quartiles were 87.3–94.3 mm Hg and >94.3 mm Hg, respectively. Participants in each DBP quartile were further grouped into intensive BP lowering and usual care groups based on the assignment in the CRHCP. The number of participants in the Q1–Q4 intervention group and usual care group was Q1: 4134, 4255; Q2: 4194, 4187; Q3: 4318, 3997; Q4: 4421, 3782, respectively (figure 1).

### Intervention and measurements

According to clinical guidelines, a stepwise management plan for hypertension was implemented to achieve the goals of SBP <130 mm Hg and DBP <80 mm Hg.^18^ The study intervention involved participants receiving a comprehensive treatment strategy from trained non-physician community healthcare providers, with a BP goal of 130/80 mm Hg; the control group received usual care. The intervention has been described in detail.^16 17^ Intervention group patients received monthly antihypertensive medications either free or at a reduced cost, along with home BP monitoring devices and had regular health coaching from the dedicated non-physician team.

Participants were followed up every 6 months after baseline enrolment. An automatic BP monitor (Omron HBP-1100U; Tokyo, Japan) was used to measure BP with one of four cuff sizes (small, standard, large or thigh) selected based on the arm circumference of each participant. During follow-up visits, after a 5 min rest, three BP measurements were taken with participants in a seated position, according to the standard protocol. Participants’ BP measurements were transmitted in real time to the research data centre through mobile devices to avoid observer bias.

### Definition of outcomes

All participants were followed up for 36 months. The major outcome was the composite of myocardial infarction, stroke, heart failure requiring hospitalisation and CVD death. We also monitored side effects to assess the safety of the intervention measures. Serious adverse events included death and hospitalisation. Other types of side effects included injurious falls, hypotension, symptomatic hypotension and syncope. We also analysed electrolyte abnormalities and renal outcomes.

### Statistical analysis

Continuous variables are expressed as mean±SD, and categorical variables are expressed as frequency (percentage). Baseline data were assessed for differences between groups using the student t-test and analysis of variance for continuous variables or the χ² test for categorical variables. The mean BP at 12 months, 24 months and 36 months for different DBP quartiles was used to compare BP changes in the different groups.

Before the construction of the Cox proportional regression models, we systematically assessed the adherence to the proportional hazards assumption. After adjusting for age, sex, smoking, use of antihypertensive drugs, history of CVD, baseline SBP, low-density lipoprotein (LDL) cholesterol and fasting blood glucose, marginal Cox proportional hazards model was used to estimate the HR and 95% CI of the primary outcome in each quartile related to intervention, setting the village as a random effect and stratification variables (province, county and township) as fixed effects. A robust sandwich covariance matrix was used to account for village clusters.^19 20^ To test the interaction effect, the multiplicative interaction term between groups and baseline DBP quartiles was additionally introduced into the regression model. We addressed any missing data using chained random forests with predictive mean matching (missRanger in R) and completed the sensitivity analysis. Kaplan-Meier survival curve analysis and Cox proportional hazards models were used to explore the cumulative incidence of outcomes in different baseline DBP quartiles. Subgroup analyses were conducted to test the robustness of the results. We also examined the impact of the achieved DBP (as a continuous variable) on outcomes by constructing a cubic spline regression model. The model accounted for randomisation and adjusted for the aforementioned covariates. In addition, we also used this model to evaluate the association between the achieved DBP and outcomes in participants with SBP of 140–159 mm Hg and DBP <90 mm Hg and with SBP ≥160 mm Hg and DBP <90 mm Hg. Unadjusted crude risk ratios were used for safety and renal outcomes. Two-tailed p values<0.05 were considered statistically significant for all analyses. Statistical analysis was conducted with SAS V.9.4 (SAS Institute, Cary, North Carolina, USA), R V.4.2.0 (R Project for Statistical Computing, Vienna, Austria) and Stata V.18.

## Results

### Baseline participant characteristics

In total, 33 288 participants were ultimately analysed in this study, with a total of 17 067 participants from intensive BP care group and 16 221 participants from usual care group. The baseline characteristics of participants in each DBP quartile were compared (table 1). The participants in the first DBP quartile had the highest mean 10-year risk for atherosclerotic CVD. Individuals with higher DBP levels were younger and also had unhealthier lifestyles, with increased rates of smoking and drinking alcohol. Although there was a significant difference between the intervention group and usual care group in terms of hypertension medication, SBP and LDL levels, the numerical difference was small.

**Table 1:** Baseline characteristics by baseline DBP quartile

Characteristics	Quartile 1			Quartile 2			Quartile 3			Quartile 4		
	≤80.7 mm Hg (n=8389)			80.7–87.3 mm Hg (n=8381)			87.3–94.3 mm Hg (n=8315)			>94.3 mm Hg (n=8203)		
	Intervention	Usual care	P value	Intervention	Usual care	P value	Intervention	Usual care	P value	Intervention	Usual care	P value
Age, years	67.9 (8.0)	68.0 (8.2)	0.587	63.5 (8.6)	63.7 (8.6)	0.307	61.1 (8.7)	61.2 (8.9)	0.465	59.1 (9.0)	59.5 (8.9)	0.029
Female sex, %	2851 (69.0)	2975 (69.9)	0.343	2680 (63.9)	2689 (64.2)	0.759	2606 (60.4)	2320 (58.0)	0.032	2268 (51.3)	2015 (53.3)	0.074
Education, n (%)			0.368			0.026			0.063			0.324
Primary school or less	3153 (76.3)	3291 (77.3)		2880 (70.5)	2952 (68.7)		2650 (61.4)	2547 (63.7)		2589 (58.6)	2262 (59.8)	
Junior high school	823 (19.9)	801 (18.8)		1057 (25.2)	1014 (24.2)		1359 (31.5)	1170 (29.3)		1499 (33.90)	1245 (32.9)	
High school	148 (3.6)	152 (3.6)		225 (5.4)	201 (4.8)		269 (6.2)	249 (6.2)		294 (6.7)	239 (6.3)	
College or higher	10 (0.2)	11 (0.3)		32 (0.8)	20 (0.5)		40 (0.9)	31 (0.8)		39 (0.9)	36 (1.0)	
Major CVD history, n (%)*	920 (22.3)	934 (22.0)	0.737	927 (22.1)	891 (22.3)	0.361	858 (19.9)	698 (17.5)	0.005	944 (21.4)	751 (19.9)	0.095
CKD, n (%)	28 (0.7)	20 (0.5)	0.208	28 (0.7)	22 (0.5)	0.398	30 (0.7)	27 (0.7)	0.915	21 (0.5)	20 (0.5)	0.730
Smoking, n (%)			0.391			0.129			0.030			0.932
Never smoked	3095 (74.9)	3148 (74.0)		3025 (72.10)	2980 (71.2)		3027 (70.10)	2707 (67.7)		2865 (64.80)	2440 (64.5)	
Former smokers	306 (7.4)	328 (7.7)		381 (9.1)	349 (8.3)		351 (8.10)	356 (8.9)		381 (8.60)	354 (9.4)	
Current smokers	733 (17.7)	779 (18.3)		788 (18.8)	858 (20.5)		940 (21.80)	934 (23.4)		1175 (26.60)	988 (26.1)	
Weekly alcohol drinking, n (%)	423 (10.2)	483 (11.4)	0.053	543 (12.9)	602 (14.4)	0.059	778 (18.0)	747 (18.7)	0.465	1013 (22.9)	825 (21.8)	0.102
Mean 10-year risk for ASCVD (SD), %†	17.3 (11.9)	17.0 (11.7)	0.324	14.3 (11.8)	13.8 (11.1)	0.123	13.0 (11.4)	13.2 (11.3)	0.499	14.0 (12.1)	13.9 (11.9)	0.674
Median duration of hypertension, years	8 (5–12)	8 (4–11)	0.038	7 (4–10)	7 (4–11)	0.280	7 (4–11)	7 (4–10)	0.034	7 (5–11)	7 (4–10)	0.001
Use of antihypertensive medications, n (%)	3306 (80.0)	3171 (74.5)	0.001	3448 (82.2)	3254 (77.7)	0.001	3462 (80.2)	3025 (75.7)	0.001	3803 (86.0)	3087 (81.6)	0.001
Pulse pressure, mm Hg	75.0 (14.52)	74.6 (14.6)	0.266	67.3 (15.42)	66.4 (15.1)	0.006	65.4 (15.6)	64.6 (15.5)	0.023	68.2 (16.8)	66.9 (16.3)	0.001
Mean arterial pressure, mm Hg	99.6 (5.9)	99.5 (5.7)	0.389	106.6 (5.4)	106.3 (5.3)	0.002	112.6 (5.6)	112.27 (5.5)	0.021	124.29 (8.9)	123.66 (8.9)	0.001
DBP, mm Hg	74.65 (5.1)	74.7 (5.14)	0.937	84.2 (1.89)	84.1 (1.9)	0.151	90.8 (2.0)	90.7 (2.0)	0.644	101.56 (6.3)	101.35 (6.3)	0.119
SBP, mm Hg	149.6 (13.9)	149.3 (13.6)	0.252	151.5 (15.5)	150.5 (15.2)	0.004	156.1 (15.7)	155.3 (15.5)	0.02	169.8 (18.7)	168.3 (18.3)	<0.001
BMI, kg/m^2^	25.3 (3.8)	25.0 (3.8)	0.001	25.9 (3.7)	25.8 (3.7)	0.054	26.08 (3.74)	26.0 (3.8)	0.441	26.7 (4.1)	26.6 (3.9)	0.076
eGFR, mL/min/1.73 m^2^‡	92.9 (12.6)	92.5 (12.6)	0.098	96.0 (12.6)	95.9 (12.0)	0.684	97.2 (12.5)	96.9 (12.3)	0.283	97.7 (13.1)	97.0 (13.4)	0.015
TC, mg/dL	5.05 (1.0)	5.0 (1.0)	0.118	5.0 (1.0)	5.0 (1.0)	0.084	5.0 (1.0)	5.0 (1.0)	0.085	5.0 (1.0)	5.1 (1.0)	0.055
FPG, mg/dL	6.3 (2.3)	6.3 (2.2)	0.983	6.2 (2.1)	6.1 (2.0)	0.082	6.1 (2.0)	6.1 (2.0)	0.124	6.0 (1.7)	6.1 (1.80)	0.871
LDL, mg/dL	2.8 (0.8)	2.7 (0.8)	0.023	2.7 (0.8)	2.7 (0.8)	0.066	2.7 (0.8)	2.7 (0.8)	0.350	2.7 (0.8)	2.7 (0.8)	0.035
HDL, mg/dL	1.46 (0.34)	1.5 (0.3)	0.931	1.4 (0.3)	1.4 (0.3)	0.212	1.5 (0.4)	1.4 (0.3)	0.003	1.4 (0.4)	1.4 (0.4)	0.019
Uric acid, mg/dL	293.2 (81.0)	294.8 (81.0)	0.373	299.0 (82.4)	299.1 (83.0)	0.856	301.0 (91.4)	305.6 (88.00)	0.018	313.1 (86.5)	313.7 (88.0)	0.749

* Major CVD history includes myocardial infarction, stroke and heart failure.

† ASCVD risk was calculated based on the American College of Cardiology and American Heart Association pooled cohort equations.

‡ eGFR was calculated based on the 2021 Chronic Kidney Disease Epidemiology Collaboration creatinine equations.

ASCVD, atherosclerotic cardiovascular disease; BMI, body mass index; CKD, chronic kidney disease; CVD, cardiovascular disease; DBP, diastolic blood pressure; eGFR, estimated glomerular filtration rate; FPG, fasting plasma glucose; HDL, high-density lipoprotein; LDL, low-density lipoprotein; SBP, systolic blood pressure; TC, total cholesterol.

### Blood pressure and medication use at the last follow-up

The BP values obtained at the end of follow-up, according to baseline DBP quartiles, are summarised in table 2. In the intervention group, both SBP and DBP decreased in 3 years of follow-up. Achieved SBP and DBP in each baseline DBP quartile in the intervention group was lower than those in the usual care group. Among the highest baseline DBP quartile of the intervention group, DBP and SBP decreased most, with decreases of 25.5 mm Hg and 43.4 mm Hg, respectively. BP control rates in the intensive treatment group were 74.2% (Q1), 75.8% (Q2), 73.3% (Q3) and 67.4% (Q4), and those in the usual care group were 33.4% (Q1), 21.2% (Q2), 14.5% (Q3) and 8.4% (Q4). Compared with the usual care group, the BP control rate in the intensive treatment group increased by nearly two to three times. Antihypertensive medication use at 36 months across different DBP quartiles is presented in online supplemental table 1, figure 1.

**Table 2:** Blood pressure at baseline and follow-up with intervention and usual care, by quartile at baseline

Time	Q1			Q2			Q3			Q4		
	Intervention	Usual care	P value	Intervention	Usual care	P value	Intervention	Usual care	P value	Intervention	Usual care	P value
DBP, mm Hg												
Baseline	74.7 (5.1)	74.7 (5.1)	0.937	84.2 (1.9)	84.1 (1.9)	0.151	90.8 (2.0)	90.7 (2.0)	0.644	101.6 (6.3)	101.3 (6.3)	0.119
Year 1	70.4 (9.1)	75.7 (9.3)	<0.001	76.4 (8.4)	82.8 (9.0)	<0.001	80.0 (9.0)	87.1 (8.9)	<0.001	84.8 (10.3)	94.1 (10.6)	<0.001
Year 2	67.7 (8.2)	74.3 (9.5)	<0.001	72.5 (7.5)	80.4 (8.9)	<0.001	75.3 (8.1)	83.8 (8.8)	<0.001	78.8 (9.4)	89.0 (10.4)	<0.001
Year 3	69.4 (8.0)	75.4 (9.6)	<0.001	72.5 (7.1)	81.0 (8.6)	<0.001	74.3 (7.1)	84.4 (8.8)	<0.001	76.0 (8.1)	89.3 (10.5)	<0.001
DBP change from baseline, mm Hg												
Year 1	−4.3 (8.7)	1.0 (8.5)	<0.001	−7.8 (8.4)	−1.3 (8.9)	<0.001	−10.7 (9.0)	−3.7 (8.7)	<0.001	−16.7 (10.5)	−7.1 (10.0)	<0.001
Year 2	−7.0 (8.1)	−0.4 (9.1)	<0.001	−11.7 (7.6)	−3.7 (8.8)	<0.001	−15.5 (8.1)	−6.9 (8.7)	<0.001	−22.8 (10.1)	−12.2 (10.6)	<0.001
Year 3	−5.4 (8.3)	0.7 (9.4)	<0.001	−11.7 (7.2)	−3.2 (8.6)	<0.001	−16.5 (7.3)	−6.3 (8.8)	<0.001	−25.5 (9.8)	−12.0 (11.0)	<0.001
SBP, mm Hg												
Baseline	149.6 (13.9)	149.3 (13.6)	0.252	151.5 (15.2)	150.5 (15.2)	0.252	156.1 (15.7)	155.3 (15.5)	0.020	169.8 (18.7)	168.3 (18.3)	0.004
Year 1	140.3 (17.4)	152.5 (18.6)	<0.001	139.4 (17.0)	152.4 (19.2)	<0.001	140.5 (17.2)	153.8 (19.0)	<0.001	144.0 (18.7)	161.3 (21.0)	<0.001
Year 2	131.7 (14.8)	147.5 (18.7)	<0.001	130.7 (14.3)	147.5 (18.4)	<0.001	131.6 (15.4)	148.3 (18.3)	<0.001	133.7 (16.7)	153.4 (20.2)	<0.001
Year 3	126.6 (11.6)	146.6 (17.9)	<0.001	125.7 (11.4)	145.7 (17.6)	<0.001	125.6 (11.1)	146.8 (18.1)	<0.001	126.2 (12.6)	151.2 (19.8)	<0.001
SBP change from baseline, mm Hg												
Year 1	−9.3 (18.7)	3.4 (18.3)	<0.001	−12.0 (18.0)	2.1 (18.2)	<0.001	−15.6 (18.6)	−1.5 (18.3)	<0.001	−25.7 (21.3)	−6.7 (19.8)	<0.001
Year 2	−17.8 (17.5)	−1.7 (18.6)	<0.001	−20.7 (18.0)	−3.0 (18.9)	<0.001	−24.5 (18.1)	−7.1 (18.6)	<0.001	−36.0 (21.5)	−14.7 (21.2)	<0.001
Year 3	−22.9 (17.1)	−2.5 (18.5)	<0.001	−25.7 (17.6)	−4.7 (18.1)	<0.001	−30.5 (17.8)	−8.4 (18.9)	<0.001	−43.4 (21.2)	−16.9 (21.6)	<0.001
BP <130/80 mm Hg												
Year 1	1120 (32.3)	292 (8.7)	<0.001	1140 (32.2)	262 (7.9)	<0.001	995 (27.2)	184 (5.9)	<0.001	712 (19.3)	78 (2.7)	<0.001
Year 2	2280 (61.9)	601 (16.1)	<0.001	2284 (61.6)	499 (13.6)	<0.001	2271 (59.1)	370 (10.7)	<0.001	1960 (50.6)	203 (6.3)	<0.001
Year 3	2810 (74.2)	565 (33.4)	<0.001	2927 (75.8)	515 (21.2)	<0.001	2923 (73.3)	410 (14.5)	<0.001	2750 (67.4)	245 (8.4)	<0.001

Continuous variables are expressed as mean (SD).

BP, blood pressure; DBP, diastolic blood pressure; Q, quartile; SBP, systolic blood pressure.

### Clinical outcomes

During a median of 36.8 months, the total number of primary outcomes was 1894 (790 in the intervention group and 1104 in the usual care group). For different baseline DBP quartiles, adjusted HRs of the primary outcome with intervention were Q1: 0.69 (95% CI 0.58 to 0.83, p*<*0.001), Q2: 0.60 (95% CI 0.50 to 0.71, p*<*0.001), Q3: 0.59 (95% CI 0.49 to 0.71, p*<*0.001) and Q4: 0.67 (95% CI 0.56 to 0.78, p*<*0.001), respectively (table 3). There was no significant difference between baseline DBP quartiles and the interaction of intervention (p for interaction=0.987). For stroke, the intervention showed a significant protective effect across all DBP levels (p<0.001). Significant protective effects for myocardial infarction were noted at Q2 and Q4 DBP levels, after adjusting for confounding factors (HR 0.53, 95% CI 0.30 to 0.94, p=0.028; HR 0.60, 95% CI 0.40 to 0.92, p=0.019). For heart failure, a protective effect was observed at the Q1 and Q3 DBP levels (p<0.05); the same effects were present at other levels but were not significant. The protective effect on CVD deaths was observed in the second (HR 0.70, 95% CI 0.50 to 1.00, p=0.049), third (HR 0.52, 95% CI 0.35 to 0.77, p=0.001) and fourth (HR 0.65, 95% CI 0.47 to 0.90, p=0.010) quartiles, after adjusting for confounding factors. For all-cause mortality, the protective effect was significant only in the fourth quartile (p<0.001, p for interaction=0.008). To test the robustness of the results, we conducted a sensitivity analysis after processing the missing data with multiple imputations (online supplemental table 2). The adjusted HR for each DBP quartile was consistent with the results of the primary analysis (online supplemental table 3).

**Table 3:** Intervention for primary outcome events, stroke, MI, HF, all-cause death and cardiovascular death, by baseline DBP quartile

Study outcomes	Intervention		Usual care		HR	P value	Adjusted HR	P value	P for interaction
	No. of events	Rate, % per year	No. of events	Rate, % per year	(95% CI)*		(95% CI)†		
The primary outcome of cardiovascular disease (MI, stroke, HF or cardiovascular disease death)									
First quartile	166	1.39	236	1.94	0.70 (0.59 to 0.84)	<0.001	0.69 (0.58 to 0.83)	<0.001	0.987
Second quartile	177	1.46	279	2.33	0.62 (0.51 to 0.74)	<0.001	0.60 (0.50 to 0.71)	<0.001	
Third quartile	169	1.35	253	2.21	0.61 (0.51 to 0.73)	<0.001	0.59 (0.49 to 0.71)	<0.001	
Fourth quartile	278	2.20	336	3.16	0.69 (0.59 to 0.81)	<0.001	0.67 (0.56 to 0.78)	<0.001	
Stroke									
First quartile	117	0.98	168	1.40	0.69 (0.56 to 0.85)	<0.001	0.70 (0.55 to 0.84)	<0.001	0.754
Second quartile	134	1.10	225	1.88	0.58 (0.47 to 0.71)	<0.001	0.57 (0.47 to 0.69)	<0.001	
Third quartile	142	1.13	221	1.84	0.61 (0.50 to 0.76)	<0.001	0.59 (0.48 to 0.73)	<0.001	
Fourth quartile	233	1.83	281	2.63	0.70 (0.58 to 0.83)	<0.001	0.67 (0.56 to 0.80)	<0.001	
MI									
First quartile	33	0.27	31	0.25	1.10 (0.69 to 1.75)	0.694	1.06 (0.66 to 1.68)	0.821	0.251
Second quartile	21	0.17	35	0.29	0.58 (0.34 to 1.00)	0.048	0.53 (0.30 to 0.94)	0.028	
Third quartile	20	0.16	18	0.15	1.00 (0.53 to 1.91)	0.994	0.97 (0.51 to 1.84)	0.929	
Fourth quartile	30	0.23	41	0.37	0.64 (0.42 to 0.98)	0.040	0.60 (0.40 to 0.92)	0.019	
HF									
First quartile	9	0.07	29	0.23	0.33 (0.16 to 0.67)	0.002	0.33 (0.16 to 0.71)	0.005	0.187
Second quartile	15	0.12	16	0.13	0.93 (0.47 to 1.86)	0.840	0.87 (0.44 to 1.75)	0.704	
Third quartile	9	0.07	17	0.15	0.53 (0.26 to 1.06)	0.072	0.45 (0.21 to 0.99)	0.046	
Fourth quartile	12	0.09	12	0.11	0.84 (0.39 to 1.80)	0.657	0.86 (0.40 to 1.87)	0.709	
Cardiovascular death									
First quartile	50	0.41	61	0.49	0.82 (0.57 to 1.18)	0.284	0.84 (0.58 to 1.21)	0.352	0.244
Second quartile	48	0.37	63	0.51	0.72 (0.51 to 1.03)	0.072	0.70 (0.50 to 1.00)	0.049	
Third quartile	33	0.26	56	0.48	0.55 (0.37 to 0.82)	0.003	0.52 (0.35 to 0.77)	0.001	
Fourth quartile	62	0.48	79	0.72	0.66 (0.49 to 0.91)	0.010	0.65 (0.47 to 0.90)	0.010	
Death from all causes									
First quartile	207	1.71	224	1.80	0.97 (0.81 to 1.17)	0.751	0.98 (0.81 to 1.17)	0.787	0.008
Second quartile	164	1.33	171	1.39	0.95 (0.79 to 1.15)	0.594	0.95 (0.79 to 1.16)	0.625	
Third quartile	144	1.13	144	1.23	0.93 (0.73 to 1.17)	0.533	0.93 (0.74 to 1.17)	0.541	
Fourth quartile	161	1.24	219	1.99	0.62 (0.51 to 0.77)	<0.001	0.63 (0.52 to 0.78)	<0.001	

* In the marginal Cox models, village was used as a random effect.

† Additionally adjusted for age, sex, smoking, use of antihypertensive drugs, history of cardiovascular disease, baseline systolic blood pressure, low-density lipoprotein cholesterol and fasting blood glucose.

HF, heart failure.DBP, diastolic blood pressure; MI, myocardial infarction.

**Figure 2:**
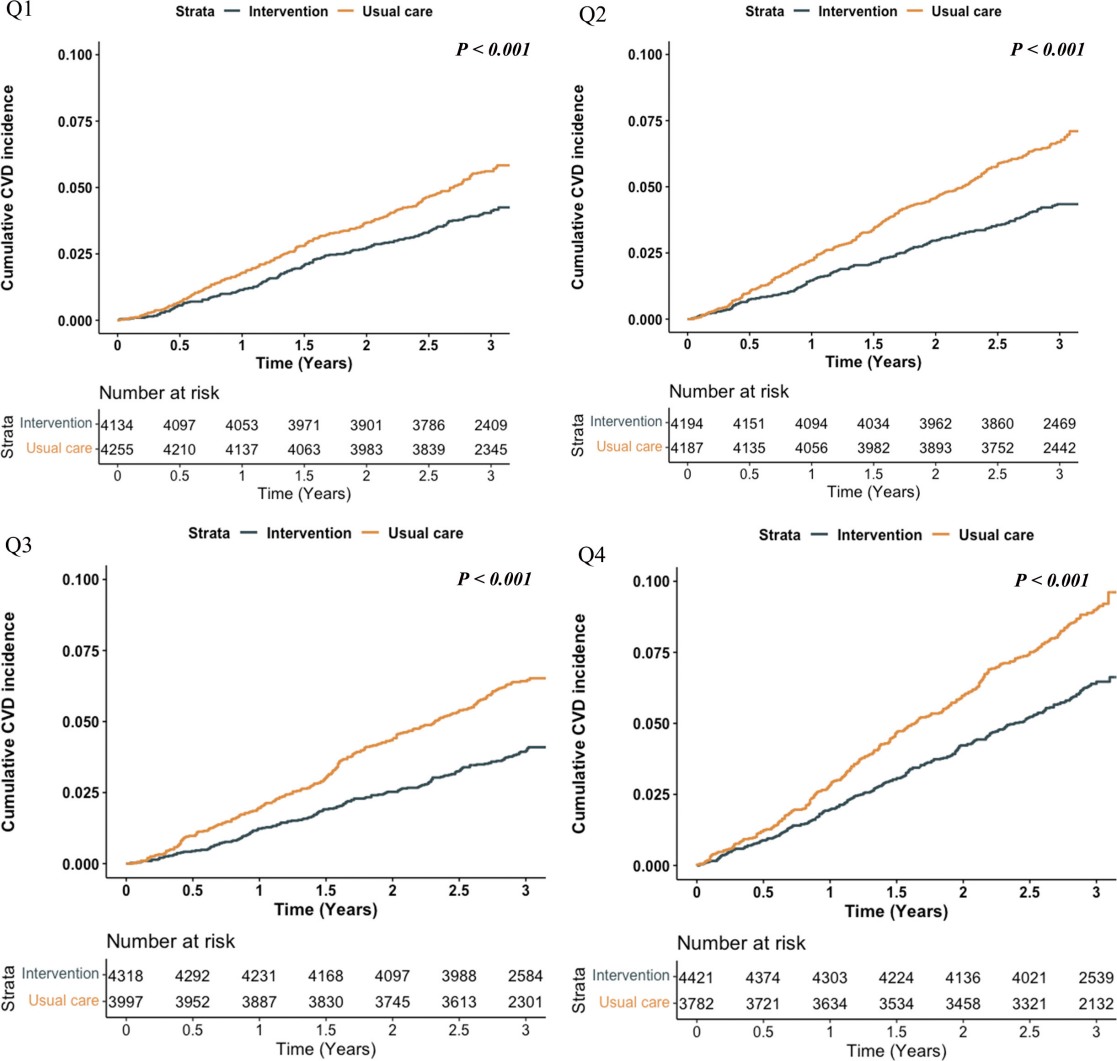
Cumulative incidence of primary outcomes for intervention versus usual care by quartiles of DBP. Cumulative incidence of cardiovascular disease in different baseline DBP quartiles Q1, Q2, Q3 and Q4, respectively. CVD, cardiovascular disease; DBP, diastolic blood pressure.

**Figure 3:**
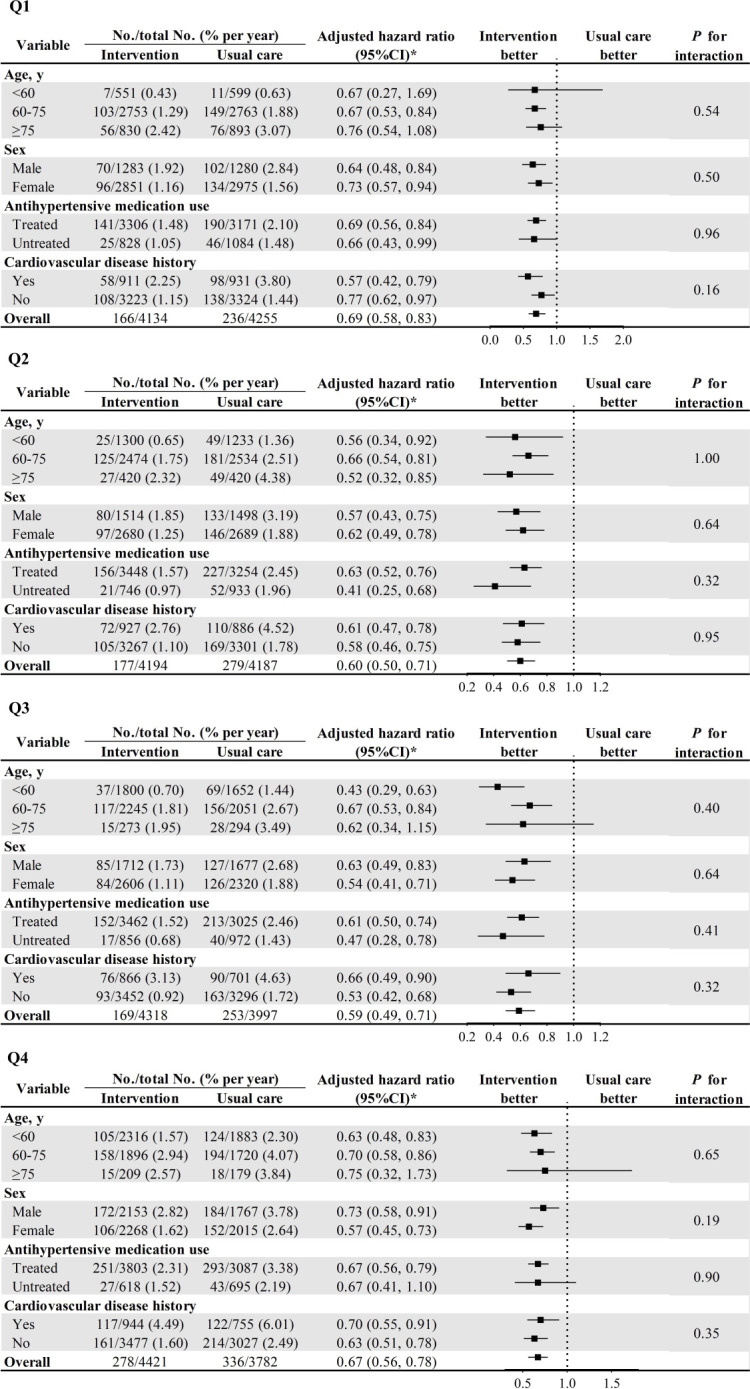
Forest plots showing results of subgroup analysis by baseline DBP quartiles. DBP, diastolic blood pressure.

Cumulative incidence curves for CVD events in the different baseline DBP groups were generated in Kaplan-Meier survival analysis (figure 2). The incidence of adverse outcomes in the intervention group was significantly lower than that in the control group, in any DBP quartile. Secondary outcomes were also assessed using Kaplan-Meier curves; the findings were consistent with those observed for the primary outcome (online supplemental figures 2–6). We performed subgroup analysis in each DBP quartile and confirmed that the model results were robust against potential confounders (figure 3).

Restricted cubic spline regression analyses relating the association between achieved DBP and cardiovascular outcomes are presented in online supplemental figure 7. Most cardiovascular outcomes (primary outcome, myocardial infarction, stroke) did not appear to be modified by the achieved DBP (all p values were >0.05). When achieved DBP ≤75 mm Hg or ≥90 mm Hg, the risk of heart failure increased (p<0.05). The same effect was observed in participants with SBP of 140–159 mm Hg and DBP <90 mm Hg, as well as SBP ≥160 mm Hg and DBP <90 mm Hg (online supplemental figures 8,9).

### Safety and renal outcomes

**Figure 4:**
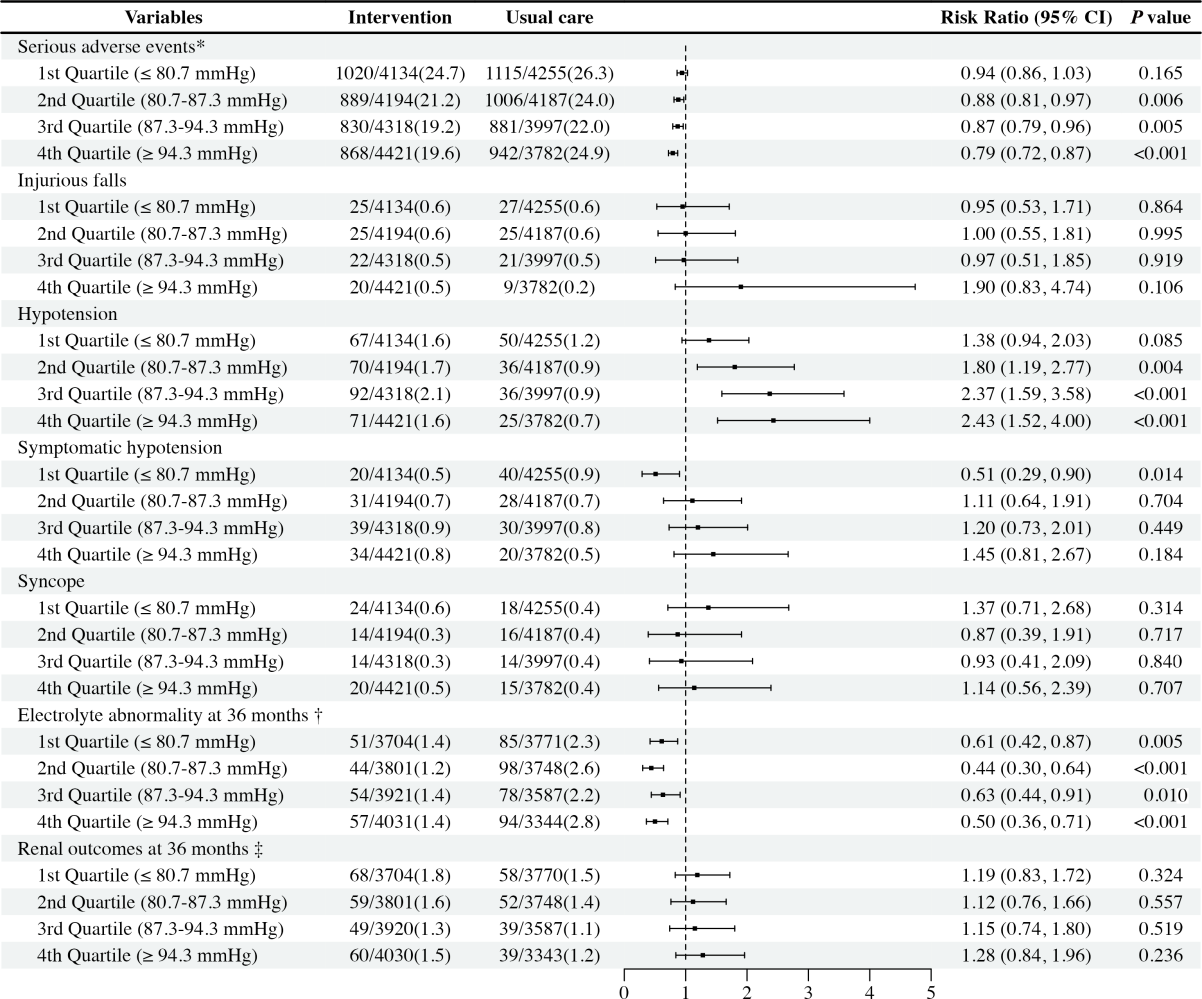
Risk ratios of intervention for adverse events by baseline DBP quartiles. *Serious adverse events include deaths and hospitalisations in this analysis. †Electrolyte abnormality at 36 months with serum sodium <130 or >150 mmol/L, or serum potassium <3.0 or >5.5 mmol/L. ‡Renal outcomes at 36 months refer to ≥50% reduction in estimated GFR in patients with chronic kidney disease at baseline, or ≥30% reduction in estimated GFR to <60 mL/min/1.73 m2 in patients without chronic kidney disease at baseline. DBP, diastolic blood pressure; GFR, glomerular filtration rate.

The incidence of safety outcomes is summarised in figure 4. In the different quartiles of baseline DBP, intervention could reduce serious adverse events including death and hospitalisation. The intervention group was more prone to hypotension, and the incidence of hypotension was higher among the participants in the third and fourth DBP quartiles compared with those in the first quartile. Intensive BP reduction did not lead to more injurious falls, syncope or adverse kidney outcomes.

## Discussion

In this study, we observed that intensive BP control had a protective effect in composite of CVD for all quartiles of baseline DBP. The protective effect showed no heterogeneity in baseline DBP quartiles.

Previously, intensive BP control strategies to mitigate the CVD risk in hypertensive patients have mainly focused on SBP; however, increasing attention has been paid to the impact of DBP on CVD outcomes. Many studies have shown that low DBP is associated with a high risk of CVD events. Farnett *et al* proposed a J-curve relationship between DBP and CVD events; patients with DBP ranging from 85 to 90 mm Hg had the maximum risk reduction, and the mortality rate owing to cardiac events increased on either side of this range.^21^ The International Verapamil-Trandolapril Study showed that as DBP decreased from 84 mm Hg, the risk of primary outcomes, all-cause death and myocardial infarction gradually increased.^10 22^ Additionally, one study found that DBP <70 mm Hg was independently associated with a general and expected increase in high sensitivity troponin T.^9^ Evidence from previous studies showed that low DBP leads to reduced myocardial perfusion in patients with coronary artery disease.^12^ However, evidence from Mendelian randomisation studies refuted the J-shaped association and established a linear correlation between DBP and CVD.^23 24^ In the current study, the restricted cubic spline analyses indicated the J-type relationship only existed in heart failure. We found that the primary outcomes, stroke and heart failure, showed significant benefits at the lowest DBP quartiles. A non-significant protective effect against other secondary outcomes such as all-cause death and CVD death existed, which may be attributed to the limited number of final outcomes. Those outcomes will continue to be monitored in ongoing follow-up of the CRHCP.

In implementation studies of intensive BP control, such as the Hypertension Optimal Treatment (HOT) study, the target DBP range was set to ≤90 mm Hg, ≤85 mm Hg and ≤80 mm Hg. Intention-to-treat analysis found no difference in CVD events, CVD mortality or all-cause mortality among the three groups.^25^ Similarly, in a post hoc analysis of the Secondary Prevention of Small Subcortical Strokes (SPS3) study, intensive BP lowering did not increase the risk of stroke in patients with lower baseline DBP.^7^ In the secondary analysis of the Systolic Blood Pressure Intervention Trial (SPRINT), with the target SBP set to 120 mm Hg, data showed that intensive control of SBP was also beneficial when the baseline DBP was at the lowest level.^14^ We obtained similar results in our study, indicating that with the support of such BP control strategies, the intervention group showed considerable benefits regardless of baseline DBP levels. Unlike the SPRINT study, the CRHCP set a target of 130/80 mm Hg (recommended in recent guidelines) as the goal of intensive BP control, which is undoubtedly safer. Our secondary analysis of CRHCP study data had a larger sample size than the SPRINT study, which excluded patients with stroke and diabetes. We included these participants as a valuable complement to SPRINT. Our findings were consistent with those of the HOT and SPRINT studies, supporting the hypothesis that lower baseline DBP does not mitigate the beneficial effects of intensive BP control in reducing CVD events. In this study, we conducted an interaction test of the treatment group across the baseline DBP quartile, which showed no heterogeneous effect. Therefore, low baseline DBP should not be an obstacle to intensive BP control in clinical practice.

In terms of safety, the intervention group exhibited a higher propensity for hypotension across all baseline DBP stratifications, aligning with the findings observed in the entire population of CRHCP. It is noteworthy that participants in the third and fourth quartiles (with higher baseline BP) exhibited a higher incidence of hypotension. This is likely attributable to a more substantial reduction in BP and the greater intensity of antihypertensive treatment (reflected in the higher proportion of multidrug combination therapy) in these patients. In all quartiles of baseline DBP, our intervention strategy could reduce serious adverse events including death and hospitalisation, but not the incidence of injurious falls, syncope or kidney outcomes. This was different from the SPRINT study and may be owing to a target BP value of 130/80 mm Hg.

This study also has several limitations. First, post hoc analysis offers less statistical power than randomised controlled trials. However, we made efforts to minimise selection bias and assessed the adjusted HR to mitigate any imbalances. Second, BP levels decreased gradually, so the effect of BP lowering would be delayed; we may not have observed sufficient outcome differences. Third, this study lacks an assessment of frailty and functional impairment, which limits the generalisability and applicability of the findings to older and more vulnerable populations. Besides, the BP values used for statistical evaluation in this study were all based on on-site measurements by community health workers, which can only be reduced but not completely eliminated the white-coat effect. The above limitations are expected to be improved in future research.

## Conclusions

In summary, our study showed that non-physician community healthcare provider-led intervention with a target of BP <130/80 mm Hg was effective and safe across all baseline DBP groups, even in the lowest baseline DBP quartile. CVD outcomes remained similar protective effects across each quartile. Thus, this effective strategy is capable of being implemented into low resource settings across the world.

## Supplementary material

10.1136/heartjnl-2025-326413 online supplemental file 1

## Data Availability

Data may be obtained from a third party and are not publicly available.
