# Supplementary material for: Efficacy and safety of intensive antihypertensive strategy in patients with low diastolic blood pressure: a secondary analysis of a cluster randomised trial
Source: Heart. 2025 Dec 9;112(15):e326413. doi: 10.1136/heartjnl-2025-326413 (PMC13479195; doi:10.1136/heartjnl-2025-326413)
Supplement: Supplementary file 1 [file heartjnl-112-15-s001.docx]

**Supplementary Appendix**

[**Table S1. Types of antihypertensive medications used at 36-month follow-up**	2](#_Toc211097788)

[**Table S2. Baseline characteristics in baseline DBP quartiles after multiple imputation**	4](#_Toc211097789)

[**Table S3. Intervention for outcome events by baseline DBP quartiles after multiple imputation**	6](#_Toc211097790)

[**Figure S1. Number of antihypertensive medications used at 36-month follow-up**	8](#_Toc211097791)

[**Figure S2. Cumulative incidence of stroke for intervention versus usual care group by quartiles of DBP**	9](#_Toc211097792)

[**Figure S3. Cumulative incidence of myocardial infarction for intervention versus usual care by quartiles of DBP**	10](#_Toc211097793)

[**Figure S4. Cumulative incidence of heart failure for intervention versus usual care by quartiles of DBP**	11](#_Toc211097794)

[**Figure S5. Cumulative incidence of cardiovascular disease death for intervention versus usual care by quartiles of DBP**	12](#_Toc211097795)

[**Figure S6. Cumulative incidence of all-cause death for intervention versus usual care by quartiles of DBP**	13](#_Toc211097796)

[**Figure S7. Adjusted restricted cubic spline analyses for achieved DBP as a continuous variable**	14](#_Toc211097797)

[**Figure S8. Adjusted restricted cubic spline analyses for achieved DBP as a continuous variable in individuals with a SBP of 140-159 mmHg and DBP <90 mmHg**	15](#_Toc211097798)

[**Figure S9. Adjusted restricted cubic spline analyses for achieved DBP as a continuous variable in individuals with a baseline SBP ≥160 mmHg and DBP <90 mmHg**	16](#_Toc211097799)

**Table S1.** **Types of antihypertensive medications used at 36-month follow-up**

| **Antihypertensive medications** | **Q1** | | **Q2** | | **Q3** | | **Q4** | |
| --- | --- | --- | --- | --- | --- | --- | --- | --- |
|  | **Intervention** | **Usual care** | **Intervention** | **Usual care** | **Intervention** | **Usual care** | **Intervention** | **Usual care** |
| No∙ of participants who reported taking antihypertensive medications during the past 2 weeks | 3728 | 3075 | 3811 | 3087 | 3925 | 2967 | 4049 | 2907 |
| Calcium channel blockers, n (%) | 3328 (89.3) | 1591 (40.0) | 3316 (87.0) | 1567 (50.8) | 3502 (89.2) | 1511 (50.9) | 3647 (90.1) | 1582 (54.4) |
| ACE inhibitors/angiotensin II receptor blockers, n (%) | 1819 (48.8) | 1011(25.4) | 1876 (49.2) | 1044 (33.8) | 1881 (47.9) | 1095 (36.9) | 1946 (48.1) | 1086 (37.4) |
| Diuretics, n (%) | 1112 (29.8) | 215(5.41) | 1273 (33.4) | 221 (7.2) | 1287 (32.8) | 231 (7.8) | 1369 (33.8) | 229 (7.9) |
| Combination of ACE inhibitors/angiotensin II receptor blockers with diuretics, n (%) | 1273 (34.2) | 91 (2.29) | 1241 (32.6) | 81 (2.6) | 1369 (34.9) | 90 (3.0) | 1518 (37.5) | 105 (3.6) |
| Beta blockers, n (%) | 45 (1.2) | 57(1.43) | 42 (1.1) | 47 (1.5) | 52 (1.3) | 56 (1.9) | 62 (1.5) | 56 (1.9) |
| Combination of ACE inhibitors/angiotensin II receptor blockers with calcium channel blockers, n (%) | 10 (0.27) | 10 (0.25) | 7 (0.2) | 10 (0.3) | 8 (0.2) | 18 (0.6) | 17 (0.4) | 16 (0.6) |
| Others, n (%) | 7 (0.19) | 357 (9.0) | 14 (0.4) | 373 (12.1) | 22 (0.6) | 275 (9.3) | 50 (1.2) | 294 (4.2) |

Angiotensin-converting enzyme (ACE) inhibitors/angiotensin II receptor blockers: benazepril, candesartan, captopril, enalapril, fosinopril, imidapril, irbesartan, lisinopril, losartan, olmesartan, perindopril, ramipril, telmisartan, and valsartan.

Beta blockers: arotinolol, atenolol, bisoprolol, carvedilol, labetalol, metoprolol, and propranolol.

Calcium channel blockers: amlodipine, benidipine, clinildipine, diltiazem, felodipine, lacidipine, lercanidipine, nifedipine, nimodipine, nitrendipine, and verapamil.

Diuretics: Furosemide, hydrochlorothiazide, indapamide, Spironolactone, and triamterene.

Combination of angiotensin-converting enzyme (ACE) inhibitor/angiotensin II receptor blocker with calcium channel blocker: amlodipine-benazepril, amlodipine-valsartan, lisinopril-amlodipine, and Olmesartan-amlodipine.

Combination of ACE inhibitor/angiotensin II receptor blocker with diuretic: benazepril-hydrochlorothiazide, enalapril-hydrochlorothiazide, irbesartan-hydrochlorothiazide, lisinopril-hydrochlorothiazide, losartan potassium-hydrochlorothiazide, olmesartan-hydrochlorothiazide, perindopril-indapamide, telmisartan-hydrochlorothiazide, and valsartan-hydrochlorothiazide.

Others: Beijing compound antihypertensive tablets (reserpine-dihydralazine-hydrochlorothiazide-triamterene), Compound reserpine tablets (reserpine-hydrochlorothiazide-dihydralazine-promethazine), Compound antihypertensive tablets (reserpine-dihydralazine-hydrochlorothiazide), Zhenju compound antihypertensive tablets (clonidine-hydrochlorothiazide), Compound triazine and rutinum tablets (reserpine-dihydralazine-hydrochlorothiazide), Compound bendazol hydrochlorothiazide capsules (reserpine-hydrochlorothiazide), Compound tetrazine reserpine tablets (reserpine-dihydralazine-hydrochlorothiazide), Compound apocynum (dihydralazine-hydrochlorothiazide), reserpine, dihydralazine, clonidine, and urapidil.

**Table S2. Baseline characteristics in baseline DBP quartiles after multiple imputation**

| **Characteristics** | **Quartile 1** | | | **Quartile 2** | | | **Quartile3** | | | **Quartile 4** | | |
| --- | --- | --- | --- | --- | --- | --- | --- | --- | --- | --- | --- | --- |
|  | **≤80.7 mmHg (n=8553)** | | | **80.7-87.3 mmHg (n=8549)** | | | **87.3-94.3 mmHg (n=8484)** | | | **>94.3 mmHg (n=8409)** | | |
|  | **Intervention** | **Usual care** | ***P*** | **Intervention** | **Usual care** | ***P*** | **Intervention** | **Usual care** | ***P*** | **Intervention** | **Usual care** | ***P*** |
| Age, y | 67.8(8.1) | 67.9(8.2) | 0.436 | 63.5(8.6) | 63.7(8.5) | 0.204 | 61.1(8.7) | 61.3(8.9) | 0.387 | 59.1(9.0) | 59.5(8.8) | 0.019 |
| Female sex, % | 2908(69.2) | 3044(70.0) | 0.409 | 2738(64.0) | 2739(64.2) | 0.856 | 2645(60.2) | 2364(57.8) | 0.028 | 2312(51.1) | 2075(53.5) | 0.029 |
| Education, n (%) |  |  | 0.519 |  |  | 0.113 |  |  | 0.076 |  |  | 0.465 |
| Primary school or less | 3208(76.3) | 3375(77.6) |  | 2939(68.7) | 3013(70.6) |  | 2691(61.2) | 2606(63.7) |  | 2643(58.4) | 2328(60.0) |  |
| Junior high school | 836(19.9) | 810(18.6) |  | 1082(25.3) | 1030(24.1) |  | 1394(31.7) | 1199(29.3) |  | 1547(34.2) | 1273(32.8) |  |
| High school | 150(3.6) | 153(3.5) |  | 227(5.3) | 206(4.8) |  | 270(6.1) | 252(6.2) |  | 298(6.6) | 244(6.3) |  |
| College or higher | 10(0.2) | 11(0.3) |  | 32(0.7) | 20(0.5) |  | 41(0.9) | 31(0.8) |  | 39(0.9) | 37(1.0) |  |
| Major CVD history, n (%) ^*^ | 936(22.3) | 959(22.1) | 0.812 | 943(22.0) | 920(21.6) | 0.589 | 872(19.8) | 720(17.6) | 0.009 | 962(21.3) | 778(20.0) | 0.172 |
| CKD, n (%) | 28(0.7) | 20(0.5) | 0.202 | 28(0.7) | 22(0.5) | 0.400 | 30(0.7) | 27(0.7) | 0.901 | 21(0.5) | 20(0.5) | 0.736 |
| Smoking, n (%) |  |  | 0.579 |  |  | 0.073 |  |  | 0.093 |  |  | 0.466 |
| Never smoked | 3154(75.0) | 3220(74.0) |  | 3083(72.0) | 3030(71.0) |  | 3076(70.0) | 2771(67.8) |  | 2919(64.5) | 2509(64.6) |  |
| Former smokers | 307(7.3) | 329(7.6) |  | 386(9.0) | 353(8.3) |  | 358(8.1) | 359(8.8) |  | 389(8.6) | 359(9.2) |  |
| Current smokers | 743(17.7) | 800(18.4) |  | 811(18.9) | 886(20.8) |  | 962(21.9) | 958(23.4) |  | 1219(26.9) | 1014(26.1) |  |
| Weekly alcohol drinking, n (%) | 428(10.2) | 490(11.3) | 0.154 | 561(13.1) | 621(14.5) | 0.149 | 800(18.2) | 768(18.8) | 0.774 | 1058(23.4) | 850(21.9) | 0.054 |
| Mean 10-year risk for ASCVD (SD), % ^†^ | 19.0(11.9) | 18.9(11.7) | 0.697 | 15.5(11.8) | 15.1(11.3) | 0.097 | 14.1(11.5) | 14.2(11.5) | 0.581 | 15.0(12.0) | 14.9(11.9) | 0.809 |
| Median duration of hypertension, years | 8(5-12) | 7(4-11) | 0.019 | 7(4-11) | 7(4-11) | 0.371 | 7(4-11) | 7(4-10) | 0.024 | 7(5-11） | 7(4-10) | 0.001 |
| Use of antihypertensive medications, n (%) | 3354(79.8) | 3245(74.6) | 0.001 | 3508(82.0) | 3323(77.8) | 0.001 | 3506(79.8) | 3093(75.7) | 0.001 | 3854(85.1) | 3177(81.8) | 0.001 |
| Pulse pressure, mmHg | 75.0(14.5) | 74.6(14.5) | 0.222 | 67.2(15.4) | 66.3(15.1) | 0.005 | 65.4(15.6) | 64.5(15.4) | 0.009 | 68.2(16.7) | 67.0(16.3) | 0.001 |
| Mean arterial pressure, mmHg | 99.7(5.9) | 99.5(5.7) | 0.334 | 106.6(5.4) | 106.2(5.3) | 0.001 | 112.6(5.5) | 112.2(5.4) | 0.008 | 124.3(8.9) | 123.7(8.9) | 0.001 |
| DBP, mmHg | 74.7(5.1) | 74.7(5.2) | 0.954 | 84.2(1.9) | 84.1(1.9) | 0.095 | 90.8(2.0) | 90.7(2.0) | 0.600 | 101.6(6.2) | 101.4(6.3) | 0.120 |
| SBP, mmHg | 149.6(13.9) | 149.2(13.6) | 0.201 | 151.4(15.5) | 150.4(15.2) | 0.003 | 156.2(15.8) | 155.3(15.5) | 0.006 | 169.8(18.7) | 168.3(18.3) | 0.001 |
| BMI, kg/m2 | 25.3(3.8) | 25.1(3.8) | 0.001 | 25.9(3.7) | 25.8(3.7) | 0.035 | 26.1(3.7) | 26.0(3.8) | 0.412 | 26.7(4.1) | 26.5(3.9) | 0.065 |
| eGFR, mL/min/1.73m2^‡^ | 92.9(12.7) | 92.5(12.6) | 0.094 | 96.0(12.5) | 95.9(12.0) | 0.641 | 97.2(12.5) | 96.9(12.3) | 0.307 | 97.6(13.2) | 97.0(13.4) | 0.030 |
| TC, mg/dL | 5.1(1.0) | 5.0(1.0) | 0.174 | 5.0(1.0) | 5.0(1.0) | 0.062 | 5.0(1.0) | 5.0(1.0) | 0.049 | 5.0(1.0) | 5.1(1.0) | 0.036 |
| FPG, mg/dL | 6.3(2.3) | 6.3(2.2) | 0.914 | 6.2(2.1) | 6.1(2.0) | 0.109 | 6.1(2.1) | 6.1(2.0) | 0.066 | 6.0(1.8) | 6.1(1.9) | 0.571 |
| LDL, mg/dL | 2.8(0.8) | 2.7(0.8) | 0.054 | 2.7(0.8) | 2.7(0.8) | 0.043 | 2.7(0.8) | 2.7(0.8) | 0.262 | 2.7(0.8) | 2.7(0.8) | 0.025 |
| HDL, mg/dL | 1.5(0.3) | 1.5(0.3) | 0.961 | 1.4(0.3) | 1.4(0.3) | 0.220 | 1.4(0.3) | 1.4(0.3) | 0.002 | 1.4(0.4) | 1.4(0.3) | 0.021 |
| Uric acid, mg/dL | 293.1(80.5) | 294.8(80.3) | 0.328 | 298.9(81.7) | 299.1(82.2) | 0.875 | 301.1(89.8) | 305.8(87.2) | 0.015 | 313.4(85.7) | 313.6(87.2) | 0.892 |

CVD, cardiovascular disease; CKD, chronic kidney disease; ASCVD, atherosclerotic cardiovascular disease; SD, standard deviation IQR, interquartile range; DBP, diastolic blood pressure; SBP, systolic blood pressure; BMI, body mass index; eGFR, estimated glomerular filtration rate; TC, total cholesterol; FPG, asting plasma glucose; LDL, low-density lipoprotein; HDL, high-density lipoprotein.

* Major CVD history includes myocardial infarction, stroke, and heart failure.

† Atherosclerotic cardiovascular disease risk was calculated based on the American College of Cardiology and American Heart Association Pooled Cohort Equations.

‡ Estimated glomerular filtration rate was calculated based on the 2021 Chronic Kidney Disease Epidemiology Collaboration creatinine equations.

**Table S3. Intervention for outcome events by baseline DBP quartiles after multiple imputation**

| **Study outcomes** | **Intervention** | | **Usual care** | | **Hazard ratio (95% CI) ^*^** | ***P* value** | **Adjusted hazard ratio (95% CI) ^†^** | ***P* value** | ***P* for interaction** | |
| --- | --- | --- | --- | --- | --- | --- | --- | --- | --- | --- |
|  | **No. of events** | **Rate, % per year** | **No. of events** | **Rate, % per year** |  |  |  |  |  |  |
| **The primary outcome of cardiovascular disease (myocardial infarction, stroke, heart failure, or cardiovascular disease death)** | | | | | | | | | |  |
| 1st Quartile | 168 | 1.39 | 244 | 1.97 | 0.69 (0.58, 0.83) | <0.001 | 0.68 (0.57, 0.82) | <0.001 | 0.846 | |
| 2nd Quartile | 180 | 1.46 | 285 | 2.35 | 0.61 (0.51, 0.73) | <0.001 | 0.59 (0.50, 0.71) | <0.001 |  |  |
| 3rd Quartile | 175 | 1.38 | 255 | 2.19 | 0.63 (0.53, 0.76) | <0.001 | 0.61 (0.51, 0.73) | <0.001 |  |  |
| 4th Quartile | 285 | 2.21 | 343 | 3.16 | 0.68 (0.60, 0.82) | <0.001 | 0.67 (0.57, 0.79) | <0.001 |  |  |
| **Stroke** |  |  |  |  |  |  |  |  |  | |
| 1st Quartile | 118 | 0.98 | 173 | 1.39 | 0.68 (0.55, 0.84) | <0.001 | 0.67 (0.54, 0.84) | <0.001 | 0.632 | |
| 2nd Quartile | 136 | 1.10 | 230 | 1.89 | 0.57 (0.47, 0.70) | <0.001 | 0.56 (0.46, 0.68) | <0.001 |  |  |
| 3rd Quartile | 147 | 1.16 | 213 | 1.83 | 0.63 (0.52, 0.78) | <0.001 | 0.61 (0.50, 0.75) | <0.001 |  |  |
| 4th Quartile | 239 | 1.85 | 286 | 2.63 | 0.70 (0.59, 0.84) | <0.001 | 0.68 (0.57, 0.81) | <0.001 |  |  |
| **MI** |  |  |  |  |  |  |  |  |  | |
| 1st Quartile | 33 | 0.27 | 33 | 0.26 | 1.05 (0.66, 1.65) | 0.842 | 0.98 (0.62, 1.57) | 0.951 | 0.295 | |
| 2nd Quartile | 21 | 0.17 | 36 | 0.29 | 0.55 (0.32, 0.95) | 0.031 | 0.51 (0.29, 0.89) | 0.018 |  |  |
| 3rd Quartile | 21 | 0.16 | 18 | 0.15 | 1.06 (0.56, 2.00) | 0.862 | 1.03 (0.55, 1.94) | 0.928 |  |  |
| 4th Quartile | 30 | 0.23 | 42 | 0.38 | 0.63 (0.41, 0.95) | 0.029 | 0.58 (0.38, 0.88) | 0.010 |  |  |
| **HF** |  |  |  |  |  |  |  |  |  | |
| 1st Quartile | 10 | 0.08 | 29 | 0.23 | 0.37 (0.18, 0.73) | 0.005 | 0.37 (0.18, 0.77) | 0.008 | 0.195 | |
| 2nd Quartile | 15 | 0.12 | 16 | 0.13 | 0.94 (0.47, 1.86) | 0.849 | 0.88 (0.44, 1.76) | 0.713 |  |  |
| 3rd Quartile | 9 | 0.07 | 17 | 0.14 | 0.50 (0.24, 1.07) | 0.073 | 0.46 (0.21, 1.00) | 0.051 |  |  |
| 4th Quartile | 13 | 0.10 | 12 | 0.11 | 0.91 (0.43, 1.92) | 0.811 | 0.94 (0.44, 1.99) | 0.867 |  |  |
| **Cardiovascular death** | |  |  |  |  |  |  |  |  | |
| 1st Quartile | 50 | 0.41 | 64 | 0.51 | 0.79 (0.56, 1.12) | 0.184 | 0.80 (0.56, 1.15) | 0.232 | 0.262 | |
| 2nd Quartile | 50 | 0.40 | 65 | 0.52 | 0.72 (0.51, 1.01) | 0.06 | 0.70 (0.50, 0.99) | 0.044 |  |  |
| 3rd Quartile | 36 | 0.28 | 56 | 0.47 | 0.60 (0.40, 0.88) | 0.01 | 0.57 (0.38, 0.84) | 0.005 |  |  |
| 4th Quartile | 64 | 0.48 | 84 | 0.75 | 0.64 (0.47, 0.88) | 0.005 | 0.64 (0.47, 0.87) | 0.005 |  |  |
| **Death from all causes** | |  |  |  |  |  |  |  |  | |
| 1st Quartile | 209 | 1.71 | 228 | 1.80 | 0.97 (0.80, 1.16) | 0.699 | 0.97 (0.81, 1.16) | 0.751 | 0.007 | |
| 2nd Quartile | 167 | 1.33 | 174 | 1.39 | 0.94 (0.78, 1.14) | 0.533 | 0.95 (0.79, 1.15) | 0.589 |  |  |
| 3rd Quartile | 148 | 1.15 | 145 | 1.21 | 0.96 (0.76, 1.21) | 0.702 | 0.96 (0.76, 1.21) | 0.750 |  |  |
| 4th Quartile | 167 | 1.26 | 229 | 2.04 | 0.62 (0.51, 0.76) | <0.001 | 0.63 (0.52, 0.77) | <0.001 |  |  |

CI, confidence interval; MI, myocardial infarction; HF, heart failure.

*In the marginal Cox models, village was used as a random effect.

†Additionally adjusted for age, sex, smoking, use of anti-hypertensive drugs, history of cardiovascular disease, baseline systolic blood pressure, low-density lipoprotein cholesterol, and fasting blood glucose.

**Figure S1. Number of antihypertensive medications used at 36-month follow-up**

**Figure S2. Cumulative incidence of stroke for intervention versus usual care group by quartiles of DBP**

Cumulative incidence of stoke in different baseline DBP quartiles Q1, Q2, Q3, and Q4, respectively.

**Figure S3. Cumulative incidence of myocardial infarction for intervention versus usual care by quartiles of DBP**

 Cumulative incidence of MI in different baseline DBP quartiles Q1, Q2, Q3, and Q4, respectively.

MI = Myocardial infarction.

**Figure S4. Cumulative incidence of heart failure for intervention versus usual care by quartiles of DBP**

Cumulative incidence of HF in different baseline DBP quartiles Q1, Q2, Q3, and Q4, respectively.

HF = heart failure

**Figure S5. Cumulative incidence of cardiovascular disease death for intervention versus usual care by quartiles of DBP**

Cumulative incidence of CVD death in different baseline DBP quartiles Q1, Q2, Q3, and Q4, respectively.
CVD = cardiovascular diseases

**Figure S6. Cumulative incidence of all-cause death for intervention versus usual care by quartiles of DBP**

Cumulative incidence of all-cause death in different baseline DBP quartiles Q1, Q2, Q3, and Q4, respectively.

**Figure S7. Adjusted restricted cubic spline analyses for achieved DBP as a continuous variable**

The association between achieved DBP modeled as a continuous variable, and the effects of intensive BP lowering was evaluated using restricted cubic splines. The analyses were adjusted for baseline characteristics including age, sex, smoking, use of anti-hypertensive drugs, history of cardiovascular disease, baseline SBP, low-density lipoprotein cholesterol, and fasting blood glucose. The dashed line indicates HR=1. Shaded areas indicate 95% CIs. The primary outcome of cardiovascular disease was a composite of myocardial infarction, stroke, heart failure, or cardiovascular disease death.

**Figure S8. Adjusted restricted cubic spline analyses for achieved DBP as a continuous variable in individuals with a SBP of 140-159 mmHg and DBP <90 mmHg**

The association between achieved DBP modeled as a continuous variable, and the effects of intensive BP lowering was evaluated using restricted cubic splines. The analyses were adjusted for baseline characteristics including age, sex, smoking, use of anti-hypertensive drugs, history of cardiovascular disease, baseline SBP, low-density lipoprotein cholesterol, and fasting blood glucose. The dashed line indicates HR=1. Shaded areas indicate 95% CIs. The primary outcome of cardiovascular disease was a composite of myocardial infarction, stroke, heart failure, or cardiovascular disease death.

**Figure S9. Adjusted restricted cubic spline analyses for achieved DBP as a continuous variable in individuals with a baseline SBP ≥160 mmHg and DBP <90 mmHg**

The association between achieved DBP modeled as a continuous variable, and the effects of intensive BP lowering was evaluated using restricted cubic splines. The analyses were adjusted for baseline characteristics including age, sex, smoking, use of anti-hypertensive drugs, history of cardiovascular disease, baseline SBP, low-density lipoprotein cholesterol, and fasting blood glucose. The dashed line indicates HR=1. Shaded areas indicate 95% CIs. The primary outcome of cardiovascular disease was a composite of myocardial infarction, stroke, heart failure, or cardiovascular disease death.
